# Never Has There Been a Shade^1^

**DOI:** 10.3201/eid1509.000000

**Published:** 2009-09

**Authors:** Polyxeni Potter

**Affiliations:** Centers for Disease Control and Prevention, Atlanta, Georgia, USA

**Keywords:** Art science connection, emerging infectious diseases, art and medicine, Abu’l Hasan, Squirrels in a Plane Tree with Hunter Attempting to Climb the Tree, Mughal Dynasty painting, Kyasanur Forest disease virus, painting of India, Emperor Jahangir, about the cover

**Figure:**
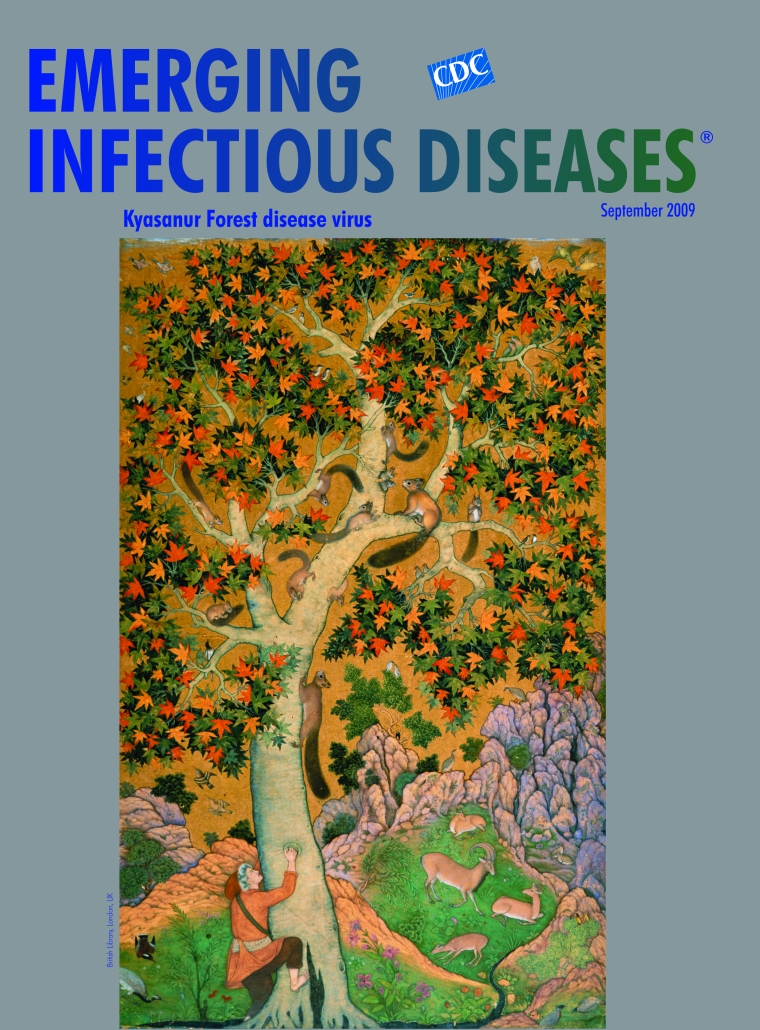
**Abu'l Hasan, Nadir al-Zaman (1588–c.1635) Squirrels in a Plane Tree with Hunter Attempting to Climb the Tree (1605–08)** Gouache on paper (22 cm × 36 cm) British Library, London, UK

“On this date [in 1618] Abu’l Hasan the artist, who had been awarded the title Nadir al-Zaman [Wonder of the Age], presented a painting he had made …. Since it was worthy of praise, he was shown limitless favor. Without exaggeration, his work is perfect, and his depiction is a masterpiece of the age.” These were the words of Emperor Jahangir of the Mughal Dynasty of India. “Abu’l Hasan’s father,” Jahangir continued, “was Aqa Reza of Herat [in western Afghanistan], who joined my service while I was still a prince. Abu’l Hasan therefore is a *khanazad* [born in the household, a second generation painter attached to the court].” His earliest known work, executed at age 12, a drawing after Albrecht Dürer’s series the Apocalypse of St. John, already showed the promise of his mature work.

The Mughal Dynasty, known for its contributions in the political unification of India, also marked a golden age for the arts; particularly during the reign of Jahangir, when art intended to document the life and culture of the court flourished and a distinctive style developed known as Mughal painting. An opinionated collector and connoisseur, Jahangir was a literate and refined man. “I derive such enjoyment from painting and have such expertise in judging it,” he wrote in his Memoirs, “that, even without the artist’s name being mentioned, no work of past or present masters can be shown to me that I do not instantly recognize who did it.”

Also a writer and naturalist, this aesthete emperor housed and recorded flora and fauna from near and afar. He took pity on elephants in winter and provided heated water for them to bathe in; he had shawls made for jackals to shield them against the cold. He encouraged detailed depictions of these and other animals, which his court painters produced prolifically, along with faithful copies of art prints, brought to India by missionaries.

During the 16th and 17th centuries, trade and the movement of humans, animals, and plants increased around the globe. Italian Renaissance reached the Mughal court, and the representational realism of the court, shaped by Hindu, Persian, and Chinese influences, found its way into European painting as Dutch and other artists visited India. Rembrandt collected Mughal works, while artists of the court copied Dürer and English painters Nicholas Hilliard and Isaac Oliver. Still, Mughal painters treaded their own path, resisting such preoccupations as perspective and the use of chiaroscuro to create the illusion of depth. Instead, they painted flat patterns, which they burnished for a jewel-like effect.

Despite Jahangir’s far-sighted insistence that paintings be cataloged, dated, and even signed, little besides names is known about the artists who served in his and other courts or about their lives and status in society. Wages were roughly equivalent to those of soldiers, though bonuses were given for outstanding work, and artists traveled with the court to wars and hunts. Not many of Abu’l Hasan’s paintings have survived, though the few that have show a variety of subjects, among them scenes of everyday life. His portraits and religious paintings were displayed in public places and were preserved in albums made for the emperor.

Squirrels in a Plane Tree, on this month’s cover, the most famous painting associated with Abu’l Hasan, shows a naturalism that must have followed direct observation. And because the common red squirrels (*Sciurus vulgaris*) in it are European natives not found in India, the artist must have observed them either in Jahangir’s zoo or abroad during one of the emperor’s travels.

Backdrop for the gamboling creatures is the iconic plane tree (*Platanus orientalis*), a fixture of the Indian countryside and a royal tree to the Mughals. This was the Tree of Hippocrates, under which the sage physician taught medicine in Kos. With a range from Iberia to the Himalaya, it is known as chenar in Persia. Xerxes fell in love with it, his sentiment immortalized in the namesake opera by George Frideric Handel: “Never was there made/a shade of a plant/dear and loving,/or more gentle.” Iqbal, poet of the East, traced the warmth of Kashmiri soil to the “blaze of chinars it nurses in its bosom.”

Abu’l Hasan captured the five-lobed leaves, whose horizontal orientation accounts for the legendary shade of the tree. These leaves, which Kasmiris dry and turn into charcoal to fuel makeshift heaters (*kangris*) carried under their tunics in winter, change from deep green to bright red in the fall, their beauty continuing to ignite the artistic imagination.

Rightfully at center stage, Abu’l Hasan’s plane tree is rooted in the bottom edge, while its canopy brushes the top of the composition. A couple of adult squirrels rein in nearly a dozen young, who cluster playfully in and around the trunk. Birds fly by, perch on the branches, or pick at the greenery on the ground, while goats frolic in the glade. Near the base, a hunter begins to climb toward the furry creatures. Like the squirrels, he does not have native features but seems extracted from some European painting of the period. The squirrels are disproportionately large; the local landscape dwarfed and compressed awkwardly around the exotic central theme.

This composition, too large to have been illustration for a manuscript, was probably derived from more than one source, its topical meaning now unknown. Some have surmised an allegorical connotation, one perhaps alluding to the adversarial relationship between humans and nature. But current concerns involving human–animal interaction and its many foibles invite even more tangible interpretations.

Abu’l Hasan’s bucolic tree teeming with small mammals and birds and surrounded by wildlife seems pertinent to a topic in this issue of Emerging Infectious Diseases: Kyasanur Forest disease and the namesake mammalian tick-borne virus, enzootic to limited geographic areas of India’s Karnataka State. The virus is transmitted by ticks between ground birds and small mammals. The recently discovered common ancestry of the Indian and Saudi Arabian Kyasanur Forest disease viruses, despite their large geographic separation, indicates long-range movement of virus, possibly by birds.

While no one knows why Abu’l Hasan’s hapless hunter was climbing the plane, any modern viewer can surmise the futility of his bare-handed endeavor against acrobatic rodents and birds. Not to mention that, unbeknownst to him, along with other hunters of tree-dwelling creatures, he is at great risk for virus infection. Still, his predicament pales beside that of virus hunters, who know as in the case of Kyasanur Forest disease virus, that obscured by unrecognized disease or cryptic enzootic cycles, elusive viruses may exist in other geographic areas and ecologic niches.
